# Body image in patients with somatoform disorder

**DOI:** 10.1186/s12888-018-1928-z

**Published:** 2018-10-22

**Authors:** M. Scheffers, H. Kalisvaart, J. T. van Busschbach, R. J. Bosscher, M. A. J. van Duijn, S. A. M. van Broeckhuysen-Kloth, R. A. Schoevers, R. Geenen

**Affiliations:** 1 Windesheim University of Applied Sciences, School of Human Movement and Education, Campus 2-6, 8017 CA Zwolle, the Netherlands; 2 Altrecht Psychosomatic Medicine, Vrijbaan 2, 3705 WC Zeist, the Netherlands; 3 Department of Psychiatry, University of Groningen, University Medical Center Groningen, P.O. Box 30.001, CC72, 9700, RB Groningen, the Netherlands; 4 Department of Sociology, University of Groningen, Grote Rozenstraat, 31 9712 TG Groningen, the Netherlands; 5 Research School of Behavioural and Cognitive Neurosciences (BCN), Interdisciplinary, Center for Psychopathology and Emotion regulation (ICPE), P.O. Box 30.001, CC72, 9700, RB Groningen, the Netherlands; 6 Department of Psychology, Utrecht University, Heidelberglaan 1, 3584CS, Utrecht, the Netherlands

**Keywords:** Body image, Somatoform disorder, Somatic symptom disorder, Dresden body image questionnaire

## Abstract

**Background:**

Although body-related problems are common in patients with somatoform disorder, research focusing on how patients with somatoform disorder perceive and evaluate their body is scarce. The present study compared differences in body image between patients with somatoform disorder and respondents from a general population sample. It also examined differences within the somatoform disorder group between men and women and between the diagnostic subgroups conversion disorder, pain disorder and undifferentiated somatoform disorder.

**Methods:**

Data were obtained from 657 patients (67.5% female) with somatoform disorder (DSM-IV-TR 300.7, 300.11, 300.81, 300.82) and 761 participants (58.6% female) from the general population. The Dresden Body Image Questionnaire (DBIQ) was used to assess body image in five domains: body acceptance, vitality, physical contact, sexual fulfilment, and self-aggrandizement. Confirmatory factor analysis and analyses of variance were performed. Since differences in age and sex were found between the somatoform disorder sample and the comparison sample, analyses were done with two samples of 560 patients with somatoform disorder and 351 individuals from the comparison sample matched on proportion of men and women and age.

**Results:**

Patients scored significantly lower than the comparison sample on all DBIQ domains. Men scored higher than women. Patients with conversion disorder scored significantly higher on vitality and body acceptance than patients with undifferentiated somatoform disorder and pain disorder.

**Conclusions:**

The mostly large differences in body image between patients with somatoform disorder and the comparison sample as well as differences between diagnostic subgroups underline that body image is an important feature in patients with somatoform disorder. The results indicate the usefulness of assessing body image and treating negative body image in patients with somatoform or somatic symptom disorder.

**Electronic supplementary material:**

The online version of this article (10.1186/s12888-018-1928-z) contains supplementary material, which is available to authorized users.

## Background

Somatoform disorder (SFD), the precursor diagnostic category of “somatic symptom disorder” [1], is characterized by persistent physical symptoms that suggest the presence of a medical condition, but cannot be adequately explained by such a medical condition, nor by the direct effects of substance use or by a mental condition [2]. A core feature of somatoform disorder and somatic symptom disorder is the problematic relation of patients with their body. Patients perceive their body as dysfunctional [3] and have difficulty not only to acknowledge and understand bodily signals in an adequate manner, but also to adapt their behavior according to these signals [4–7]. Core problems of SFD include distrust and non-acceptance of the body, intimacy problems, changed physical identity, loss of vitality, as well as lack of awareness and incorrect interpretation of bodily signals [6, 8–10]. All of these aspects may have substantial consequences for an individual’s development and quality of life [11]. Patients with SFD have been suggested to be impaired in “embodied mentalization”, described as “the capacity to see the body as the seat of emotions, wishes, and feelings and the capacity to reflect on one’s own bodily experiences and sensations and their relationships to intentional mental states in the self and others” ([12], p3).

Although body-related problems are common in patients with SFD, research focusing on how patients with SFD perceive and evaluate their body is scarce. A first condition for research is the possibility to assess the complex relation with their body in patients with SFD. This is important to acquire knowledge about the specificity and severity of body-related problems in patients with SFD as compared to reference groups. Moreover, specific symptoms such as pain, fatigue, or dissociation differ among diagnostic categories of SFD, and it could be studied whether their impact on the relation with one’s body differs as well [13, 14]. Finally, body-related assessment is needed as an evaluation tool when body-oriented interventions are part of the combined treatment package offered to patients with SFD [15, 16]. Thus, an adequate instrument to assess and evaluate the severity and scope of problems related to body image in people with SFD is a necessity.

In general, the term ‘body image’ has been used to describe and assess a variety of body-related phenomena, including perceptions, cognitions, and affects with regard to the body [17, 18]. However, most questionnaires measuring body image either emphasize physical appearance and weight or shape-related themes or specifically evaluate body image problems in eating disorders or body dysmorphic disorder, which makes them not particularly suitable for patients with SFD (for an overview, see [17]). Questionnaires directed at the general population mostly focus on a specific aspect of body image, such as satisfaction with body parts and processes [19, 20] or sociocultural attitudes towards appearance [21, 22]. Other questionnaires, developed for clinical use, focus on physical symptoms [23–25] or body awareness [26–28]. In SFD patients, however, all of these body-related aspects are important [5] and a self-report questionnaire addressing a broad range of body-related aspects is needed for both research and clinical practice.

For this purpose, the present study employed the Dresden Body Image Questionnaire (DBIQ) to measure a broad range of body-related self-perceptions in five domains: body acceptance, vitality, physical contact, sexual fulfilment, and self-aggrandizement [29, 30]. Especially the incorporation of physical contact and sexual fulfilment, often reported by patients as problematic topics but rarely included in questionnaires, makes the DBIQ a suitable instrument for the SFD population. One of the present study’s aims was to obtain more information on the severity of disturbances in these domains by comparing patients with SFD with a sample matched on sex and age from the general population described in an earlier study [31].

Studies of body image in the general population indicate that men and women appreciate their body image differently [32–34]. Women are generally more preoccupied and dissatisfied with their body than men [35], which may be explained by sociocultural values, genetic differences and differences in bodily development and experiences like trauma [36]. We expect these differences to be also present in the group of patients with SFD.

Body image may also differ between patients with different diagnostic categories conversion disorder, pain disorder and undifferentiated somatoform disorder. With no previous studies available, we base our expectation that patients with pain and undifferentiated somatoform disorder score lower on vitality than patients with conversion disorder on clinical observation.

In order to obtain insight into the significance of body image assessments for patients with SFD, the present study aimed to evaluate differences in body image as measured with the DBIQ between patients with SFD and a sample from the general population. It also aimed to evaluate, within the patient group, differences between women and men and between the diagnostic categories conversion disorder, pain disorder, and undifferentiated somatoform disorder. Prior to the evaluation of differences, measurement invariance across clinical and non-clinical samples and across sex in the somatoform sample was tested, in order to affirm whether comparisons are valid.

## Methods

### Participants

Participants were patients with severe SFD referred to Altrecht Psychosomatic Medicine, a tertiary care centre for psychosomatic medicine that is specialized in the treatment of patients with severe SFD. This centre is located in Zeist, the Netherlands. On average, patients admitted to this institution have had medically unexplained symptoms for 10 years and have, received five previous treatments for somatoform disorder in primary or secondary care. In about half of the cases, patients have comorbid disorders; mainly other somatoform diagnoses but also mood and anxiety disorders, substance dependence and personality disorder [37]. The main treatment criterion applied by the institution is the presence of a diagnosis of SFD (pain disorder, conversion disorder or undifferentiated SFD) as the primary disorder, in line with the criteria described in the Diagnostic and Statistical Manual of Mental Disorders (DSM-IV-TR) [2], diagnosed by a trained psychologist, and confirmed by the resident psychiatrist. Exclusion criteria applied by the treatment centre were people with (a) a diagnosis of hypochondriasis or body dysmorphic disorder, (b) a diagnosis of addiction, bipolar disorder, or psychosis, and (c) a crisis situation requiring immediate attention (e.g., high suicidality); and (d) patients under treatment by a specialized physician outside the center.

In an intensive intake procedure, all patients consecutively referred in the period 2011–2014 were assessed for eligibility for treatment. Treatment inclusion was based on an initial diagnostic assessment and on the patient’s informed consent to accept the treatment offered. All patients eligible for treatment were included in the study unless informed consent to participate in the study was not obtained.

Data were gathered from 657 patients with SFD between 24 and 69 years of age (Mean = 43.3, *SD* = 10.8), 443 women and 214 men with mean ages of 42.7 (*SD* = 11.0) and 44.5 (*SD* = 10.3) years. Table 1 shows the primary diagnoses according to DSM-IV-TR. The number of patients with conversion disorder was relatively high (22.4%) since the treatment centre is the only institute in the Netherlands with clinical facilities that admits patients that are difficult to treat in secondary care.

**Table 1:** Primary diagnoses of participants with somatoform disorder

Diagnoses^a^	*n* (%)	% men
Conversion Disorder (300.11)	147 (22.4)	37.4
Pain Disorder (307.80, 307.89)	185 (28.2)	38.9
Undifferentiated SFD (300.82)	325 (49.5)	27.4
Total	657 (100)	32.5

^a^ Diagnosis according to DSM-IV-TR

A convenience sample from the general population [31] was used as comparison. This sample consisted of 761 adults (433 women, 326 men, two persons with sex unknown), with a mean age of 30.9 years (SD = 13.6, range 18–65). Details about recruitment of participants, data collection, and measurements used can be found in [31].

### Measures

The Dresden Body Image Questionnaire (DBIQ) [29, 30] is a 35-item questionnaire with positively and negatively worded statements across five subscales: body acceptance (e.g., “I wish I had a different body”), vitality (e.g., “I am physically fit”), physical contact (e.g., “Physical contact is important for me to express closeness”), sexual fulfilment (e.g., “I am very satisfied with my sexual experiences”), and self-aggrandizement (e.g., “I use my body to attract attention”). The level of agreement with items is scored on a 5-point Likert scale ranging from 1 (= not at all) to 5 (= fully).

In a German non-clinical sample [30] Cronbach’s *α* for the subscales varied from α = .81 for self-aggrandizement to *α* = .94 for vitality. Correlations between the subscales varied from *r* = .37 (sexual fulfilment and self-aggrandizement) to *r* = .65 (body acceptance and vitality). The five-factor structure of the non-clinical sample was replicated using a confirmatory factor analysis in a clinical psychiatric sample of 560 patients, of whom 45% had somatoform complaints (CFI = .90; RMSEA = .06) [29]. In this clinical sample Cronbach’s α for the subscales varied from *α* = .83 for self-aggrandizement to *α* = .92 for sexual fulfilment. Correlations between the subscales varied between *r* = .31 (vitality and physical contact) to *r* = .65 (physical contact and sexual fulfilment).

Confirmatory factor analyses of the Dutch version of the DBIQ (DBIQ-35-NL) in the sample that was used in the present study for comparison showed a five-factor structure in accordance with the original scale, where model fit was improved significantly by moving one item from the subscale body acceptance to the subscale self-aggrandizement [31]. The equivalence of the measurement model across sex and age was evaluated in this study as well, demonstrating partial strong invariance. Internal consistency of the subscales in this Dutch version was good: Cronbach’s *α* varied from *α* = .74 for the subscale physical contact to *α* = .91 for the subscale sexual fulfilment. The correlations between the subscales varied from *r* = .17 for vitality and physical contact to *r* = .53 for acceptance and sexual fulfilment. Temporal stability over 2 weeks was satisfactory, varying from an intra-class correlation coefficient (ICC) of .64 for physical contact to .82 for vitality (see Additional file 1: Table S1 for DBIQ items in English).

### Procedure

Patients completed the Dutch version of the DBIQ as part of a routine initial diagnostic screening and provided written informed consent for the use of the data for scientific purposes. This part of the study protocol was approved by the institutional review board (CWO) of Altrecht, Zeist, the Netherlands (CWOnr 1419).

The study in the general population used as a comparison sample was conducted in agreement with the VU University Amsterdam guideline for research for educational purposes, allowing students to collect data with the use of questionnaires in healthy groups of respondents when participation is voluntary and data are analyzed anonymously. The Medical Ethics Review Committee of VU University waived the requirement for formal ethical approval of the procedures used (for more details see [31]).

### Data analysis

The factor structure of the clinical sample was evaluated using confirmatory factor analysis with maximum likelihood estimation robust to non-normality (MLR). Moreover, measurement invariance was examined across the two groups (somatoform disorder and general population) and across sex within the somatoform group, to ensure meaningful comparisons between scores in these groups [38–40]. We used the procedures and fit indices used in the study of the comparison sample [31]: model selection was performed by testing invariance by the Scaled Difference in Chi-Squares (SDCS) test [41] for nested models estimated with MLR. Because little consensus exists with regard to recommended fit indices [38], standardized root mean square residual (SRMR) and Tucker Lewis index (TLI) are reported, in addition to the comparative fit index (CFI) and root mean square error of approximation (RMSEA). Analyses were conducted with Mplus Version 5.1 [42].

SPSS 20.00 for Windows was used to compare group differences in the clinical sample with analysis of variance. Because of the differences in sample size in the diagnostic categories, Hochberg’s GT2 test was used for post hoc analyses [43]. Mean differences between subgroups were expressed in Cohen’s *d* and considered large if ≥0.80, moderate between 0.50 and 0.80 and small between 0.20 and 0.50 [44].

For comparison of the DBIQ scores across samples, the clinical sample was matched to the comparison sample on sex and age (see Additional file 2: Figure S1 for age distribution of males and females in the clinical sample and in the comparison sample). The exact matching procedure from the R package MatchIt [45] was used to make 72 groups with respondents of both groups with equal age and proportion of men and women. A total of 580 patients from the somatoform sample (387 women; 193 men) were matched to 341 respondents in the comparison sample (201 women, 140 men), with appropriate weights [46]. The weighted mean ages were 44.8 for men (range 25–65) and 42.8 for women (range 24–64) in both matched samples, with almost equal (weighted) standard deviations of 10.4 and 10.9 for men and women respectively across the two samples. Note that the matching procedure led to discarding the older respondents in the somatoform sample, whereas the younger respondents from the comparison sample were not included in the matched sample.

## Results

### Measurement invariance

CFA in the somatoform sample showed the earlier found five-factor structure, with the same item shifted as in the general population sample [31]. Evaluation of measurement invariance for the somatoform sample and the comparison sample showed a model with partial strong measurement invariance, with different loadings across the groups for items 1 (“I move gracefully”) of the subscale self-aggrandizement and item 7 (“There are lots of situations in which I feel happy about my body”) of the subscale body acceptance estimated freely, as best fit (RMSEA (90% CI) = .061 (.059–.063), SRMR = .074, CFI = .828, TLI = .823).

In the evaluation of the somatoform sample for measurement invariance with sex as a grouping variable, item 15 of the subscale body acceptance (“I choose clothing that hides the shape of my body”) was the only item not showing invariance (RMSEA (90% CI) = .061 (.058–.064), SRMR = .073, CFI = .832, TLI = .828). This item was also identified as non-invariant in the general population sample [31]. For detailed analysis of measurement invariance see Additional file 3: Table S2. Based on these analyses and based on comparisons of the scores with and without the items that are not invariant across groups, which led to only marginally different (sub)scale scores (for details see Additional file 4: Table S3), we concluded that use of the full scale ensures meaningful comparisons within this study and with results of other studies.

### Internal consistency and correlations between subscales

In the group of patients with SFD, Cronbach’s *α* for the subscales were .78 for physical contact and self-aggrandizement, .80 for vitality, .84 for acceptance and .92 for sexual fulfilment. Correlations between the subscales varied from *r* = .14 (vitality and physical contact) to *r* = .50 (self-aggrandizement and sexual fulfilment).

### Differences between SFD diagnostic categories

Table 2 shows means of the diagnostic categories for the total score and all subscales of the DBIQ. Analysis of variance of the three diagnostic categories (conversion disorder, undifferentiated SFD and pain disorder) showed statistically significant higher scores for patients with conversion disorder on overall body image, vitality and body acceptance than for patients with undifferentiated SFD and pain disorder. Differences were largest for vitality.

**Table 2:** Means (*M*) and standard deviations (*SD*) of scores on the Dresden Body Image Questionnaire (DBIQ) in subgroups of patients in three diagnostic categories of somatoform disorder, test of the difference between diagnostic categories

	Conversion Disorder (*n* = 147)	Pain Disorder (*n* = 185)	Undifferentiated SFD (*n* = 325)		
(sub) scale	*M (SD)*	*M (SD)*	*M (SD)*	*F(2)*	*p*
total score	2.78_a,b_ (0.65)	2.55_b_ (0.54)	2.60_a_ (0.56)	7.32	.001
vitality	2.56_a,b_ (0.84)	2.21_b_ (0.67)	2.07_a_ (0.62)	25.08	<.001
body acceptance	3.25_a,b_ (0.97)	2.86_a_ (0.83)	2.99_b_ (0.98)	7.00	.001
sexual fulfilment	2.61 (1.18)	2.42 (0.97)	2.49 (0.98)	1.44	.24
physical contact	3.32 (0.82)	3.19 (0.79)	3.31 (0.82)	1.47	.23
self-aggrandizement	2.31 (0.67)	2.20 (0.63)	2.27 (0.63)	1.19	.43

^a, b^Means in a row sharing subscripts are significantly different based on Hochberg’s GT2 test

### Differences between women and men

In Table 3 means of women and men with SFD on DBIQ total score and on all subscales are presented. Analysis of variance showed that men scored significantly higher than women on total DBIQ, body acceptance, sexual fulfilment and self-aggrandizement. No such differences were apparent for vitality and physical contact.

**Table 3:** Means (*M*) and standard deviations (*SD*) of scores on the Dresden Body Image Questionnaire (DBIQ) of women and men, test of the difference between women and men in the SFD sample

	women (*n* = 443)	men (*n* = 214)			
(sub) scale	*M* (*SD*)	*M* (*SD*)	*t*	*p*	Cohen’s *d*
total mean score	2.55 (0.56)	2.73 (0.61)	4.69	<.001	0.31
vitality	2.18 (0.68)	2.29 (0.78)	1.85	.07	0.15
body acceptance	2.88 (0.98)	3.12 (0.89)	5.50	<.001	0.26
sexual fulfilment	2.39 (0.99)	2.71 (1.06)	3.79	<.001	0.31
physical contact	3.25 (0.80)	3.33 (0.84)	1.08	.29	0.10
self-aggrandizement	2.20 (0.63)	2.39 (0.65)	3.70	<.001	0.30

### Comparisons of the matched samples

Table 4 presents means of DBIQ total score and subscales in the clinical and comparison sample matched on age and sex. Patients with SFD scored significantly lower (*p* < .001) than the comparison sample on DBIQ total mean score and on all subscales, with the largest differences for sexual fulfilment (1.2 point) and vitality (1.6 point). Cohen’s *d* was large (≥ 0.80) for all (sub)scales but physical contact.

**Table 4:** Means (M), standard deviations (*SD*), test of the difference (*t*), and effect size (Cohen’s *d*) of scores on the Dresden Body Image Questionnaire in age and sex matched samples of patients with somatoform disorder (*n* = 580) and comparison sample (*n* = 341)

	Somatoform	Comparison sample		
(sub)scale	*M (SD)*	*M (SD)*	*t*	Cohen’s *d*
total mean score	2.62 (0.58)	3.59 (0.42)	−29.3*	− 1.9
vitality	2.20 (0.71)	3.79 (0.58)	−36.9*	− 2.4
body acceptance	3.00 (0.94)	3.81 (0.66)	−15.2*	− 1.0
sexual fulfilment	2.48 (1.02)	3.71 (0.67)	−22.1*	− 1.4
physical contact	3.28 (0.82)	3.73 (0.58)	−9.7*	− 0.6
self-aggrandizement	2.26 (0.65)	3.00 (0.54)	−18.9*	− 1.2

*p < .001

## Discussion

The aim of the current study was to gain more detailed insight into body image in patients with SFD. To this end, we compared DBIQ scores in patients with SFD and people from the general population. In addition, we compared DBIQ scores in patients with different SFD diagnoses and scores demonstrated by women and men with SFD. After measurement invariance was confirmed across the clinical sample and the comparison sample as well as across sex in the clinical sample, the most prominent finding was that body image scores of patients with SFD were substantially lower than body image scores in the general population, showing large differences between groups on all domains of body image.

With respect to diagnostic categories of SFD, patients with conversion disorder scored higher on vitality, body acceptance and the total DBIQ score than patients with undifferentiated SFD and pain disorder. This difference in vitality scores is in accordance with our clinical impression that fatigue is less prevalent in conversion disorder. The higher score in body acceptance of patients with conversion disorder were unexpected. Patients with conversion disorder still scored substantially lower than the comparison group on all body image domains.

As hypothesized on the basis of results in non-clinical samples [35], women in the SFD sample scored lower than men on total DBIQ, body acceptance, sexual fulfilment and self-aggrandizement. No differences between women and men for vitality and physical contact were measured, which for vitality is in agreement with observations in chronic fatigue syndrome [47]. Overall, our study confirms that account should be taken of differences between men and women when assessing body image.

When tentatively comparing our findings with studies of the DBIQ in patients with mixed mental disorders [48], women with childhood trauma [49], and patients with depressive disorder [50], especially the relatively low scores on vitality for patients with somatoform disorder are noteworthy. Scores on sexual fulfilment and self-aggrandizement tend to be lower than those of the mental disorders group [48] but higher than the scores of the childhood trauma group [49], while scores on body acceptance and physical contact are about the same as in the mixed mental disorders group. Overall, body image scores appear to be about similar to scores of a sample of patients with mixed mental disorders, with lower vitality scores as the most distinct main outstanding feature in patients with somatoform disorder, especially in patients with pain disorder and undifferentiated somatoform disorder. While body-oriented psychotherapy is considered important in both severe somatoform disorder [51] and other severe mental disorders [52], the current study adds that a focus on body image might be an important aspect of these therapies.

The DBIQ covers five body-related aspects, that all proved to be substantially affected in patients with SFD. This finding, together with the evidence for partial strong measurement invariance across the comparison group and the SFD group, leads to the conclusion that the DBIQ is a suitable instrument to evaluate the broad scope of body-related problems in patients with SFD [5]. However, it should be acknowledged that the DBIQ does not cover all body-related themes. For example, body awareness, the sensory awareness that originates from the body’s physiological states, processes, actions and functions [27], is considered pivotal in the development and progress of SFD [53, 54] because lack of body awareness may undermine healthy behavior [55]. Furthermore, a self-report questionnaire such as the DBIQ does not address behavioural aspects, such as movement patterns and levels of activity [56]. Notwithstanding these restrictions, the large differences between patients with SFD and the general population comparison group on a broad range of body-related topics as well as the differences between diagnostic categories indicate the relevance of the DBIQ for patients with SFD.

Because data on the validity of the DBIQ scales are still scarce, comparisons with other assessments may be useful to support validity. The subscale vitality has an effect size (d = 2.5) comparable with that of the fatigue scale of the Checklist Individual Strength (CIS-20R) that has been used to compare patients with chronic fatigue syndrome (CFS) and a healthy reference group (*d* = 3.0) [47]. Furthermore, symptoms measured with the Symptom Checklist (SCL-90, [57]) in a severe SFD group have shown, when compared with a general population group, effect sizes that are comparable with or even smaller than those found for some DBIQ subscales (0.9 for anxiety, 1.2 for depression, 1.6 for somatization and 1.3 for overall psychopathology [51].

Future studies must establish the clinical relevance of using DBIQ scales for patients with SFD by examining the effects of treatment on body image (sensitivity to change) as well as the prognostic value of the DBIQ for treatment outcome in patients with SFD. Treatment for patients with SFD aims at goals such as reducing or coping with physical complaints, enhancing body acceptance, and ameliorating quality of life, all depending on individual situations and patient preferences. With respect to these goals, vitality and body acceptance seem to be the most relevant subscales of the DBIQ, but the current study shows that domains of self-aggrandizement, physical contact, and sexual fulfilment should not be overlooked in the assessment, treatment and evaluation of patients with SFD. In addition to its potential diagnostic importance and use in treatment evaluation, measuring body image with the DBIQ may also be valuable in clinical practice to recognize body-related themes underlying symptom presentation [58] and to enhance communication between patient and therapist about body-related experiences. Sexual fulfilment, for example, may be hampered by physical complaints [59] and is in fact, as the current study indicates, a prevalent problem for SFD patients. Because sexuality is a sensitive subject to discuss for patients as well as therapists, incorporating the domain of sexuality into a questionnaire may shed further light on possible problems with sexuality and enhance communication about this subject [60].

One of the present study’s strengths lies in the fact that its sample of patients with a certified diagnosis of severe and chronic SFD was large: this enabled us to compare body image between different SFD diagnoses as well as between patients and a sample from the general population. A limitation with respect to generalizability is that the results apply to a group that was referred to tertiary care; results cannot be generalized to patients with somatoform disorder who present themselves in secondary and primary care. The relatively high amount of comorbid disorders may have confounded the results but comorbid mental disorders are a characteristic of this group with severe somatoform disorders.

## Conclusion

The observed mostly large differences in body image between patients with somatoform disorder and the comparison sample as well as differences between diagnostic subgroups underline that body image is an important feature in patients with somatoform disorder. The results indicate the usefulness of assessing body image and treating negative body image in patients with somatoform or somatic symptom disorder.

## Additional files

Additional file 1: **Table S1.** Item means and standard deviations of the DBIQ items* in SFD sample grouped per subscale. (DOCX 22 kb)

Additional file 2: **Figure S1.** Age distribution of males and females across the three diagnostic categories and in the general population. (DOCX 99 kb)

Additional file 3: **Table S2.** Measurement invariance across the control group and the somatoform group and within the somatoform group across sex. (DOCX 21 kb)

Additional file 4: **Table S3.** Mean (M) and standard deviations (SD) of scores on the Dresden Body Image Questionnaire (DBIQ) in patients with somatoform disorder (*n* = 657) and control sample (*n* = 761), test of the difference based on scale items deleted and effect size (Cohen’s d). (DOCX 20 kb)

## Abbreviations

CFA: Confirmatory Factor Analysis
CFI: Comparative Fit Index
CFS: Chronic Fatigue Syndrome
CI: Confidence Interval
CIS: Checklist Individual Strength
CWO: Commissie Wetenschappelijk Onderzoek (institutional review board)
DBIQ: Dresden Body Image Questionnaire
DSM-IV-TR: Diagnostic and Statistical Manual of Mental Disorders, Text Revision
M: Mean
RMSEA: Root Mean Square Error of Approximation
SCL: Symptom Checklist
SD: Standard Deviation
SDCS: Scaled Difference in Chi-Squares
SFD: Somatoform Disorder
SRMR: Standardized Root Mean square Residual (SRMR)
TLI: Tucker Lewis Index

## References

[1] American Psychiatric Association (2013). Diagnostic and statistical manual of mental disorders.

[2] American Psychiatric Association (2000). Diagnostic and statistical manual of mental disorders DSM-IV-TR (text revision).

[3] Röhricht F (2011). Das theoretische Modell und die therapeutischen Prinzipien/Mechanismen einer integrativen Körperpsychotherapie (KPT) bei somatoformen Störungen. Psychother-Wiss.

[4] Creed F, Henningsen P, Fink P (2011). Medically unexplained symptoms, somatisation and bodily distress. Developing better clinical services.

[5] Henningsen P, Zipfel S, Herzog W (2007). Management of functional somatic syndromes. Lancet.

[6] Kalisvaart H, van Broeckhuysen S, Buhring M, Kool MB, van Dulmen S, Geenen R (2012). Definition and structure of body-relatedness from the perspective of patients with severe somatoform disorder and their therapists. PLoS One.

[7] Lind AB, Delmar C, Nielsen K (2014). Searching for existential security: a prospective qualitative study on the influence of mindfulness therapy on experienced stress and coping strategies among patients with somatoform disorders. J Psychosom Res.

[8] Bakal D, Coll P, Schaefer J (2008). Somatic awareness in the clinical care of patients with body distress symptoms. Biopsychosoc Med.

[9] Gard G (2005). Body awareness therapy for patients with fibromyalgia and chronic pain. Disabil Rehabil.

[10] McWhinney IR, Epstein RM, Freeman TR (1997). Rethinking somatization. Ann Intern Med.

[11] Cash TF (2004). Body image: past, present, and future. Body Image.

[12] Luyten P, van Houdenhove B, Lemma A, Target M, Fonagy P (2013). Vulnerability for functional somatic disorders: a contemporary psychodynamic approach. J Psychother Integr.

[13] Sertoz OO, Doganavsargil O, Elbi H (2009). Body image and self-esteem in somatizing patients. Psychiatry Clin Neurosci.

[14] Afrell M, Biguet G, Rudebeck CE (2007). Living with a body in pain - between acceptance and denial. Scand J Caring Sci.

[15] Joraschky P, Pöhlmann K (2014). Schatten im Körperbild. Die Bedeutung von Traumatisierungen und strukturellen Störungen. Psychodyn Psychother.

[16] Zipfel S, Herzog W, Kruse J, Henningsen P (2016). Psychosomatic medicine in Germany: more timely than ever. Psychother Psychosom.

[17] Cash TF, Smolak L (2011). Body image: a handbook of science, practice and prevention.

[18] Röhricht F, Seidler KP, Joraschky P, Borkenhagen A, Lausberg H, Lemche E, Loew T (2005). Consensus paper on the terminological differentiation of various aspects of body experience. Psychother Psychosom Med Psychol.

[19] Mendelson BK, Mendelson MJ, White DR (2001). Body-esteem scale for adolescents and adults. J Pers Assess.

[20] Secord PF, Jourard SM (1953). The appraisal of body-cathexis: body-cathexis and the self. J Consult Psychol.

[21] Fisher E, Dunn M, Thompson JK (2002). Social comparison and body image: an investigation of body comparison processes using multidimensional scaling. J Soc Clin Psychol.

[22] Heinberg LJ, Thompson JK, Stormer S (1995). Development and validation of the sociocultural attitudes towards appearance questionnaire. Int J Eat Disord.

[23] de Waal MW, Arnold IA, Spinhoven P, Eekhof JA, Assendelft WJ, van Hemert AM (2009). The role of comorbidity in the detection of psychiatric disorders with checklists for mental and physical symptoms in primary care. Soc Psychiatry Psychiatr Epidemiol.

[24] Kerns RD, Turk DC, Rudy TE (1985). The west haven-Yale multidimensional pain inventory (WHYMPI). Pain.

[25] Vercoulen JH, Swanink CM, Fennis JF, Galama JM, van der Meer JW, Bleijenberg G (1994). Dimensional assessment of chronic fatigue syndrome. J Psychosom Res.

[26] Kolk AM, Hanewald GJ, Schagen S, Gijsbers van Wijk CM (2003). A symptom perception approach to common physical symptoms. Soc Sci Med.

[27] Mehling WE, Gopisetty V, Daubenmier J, Price CJ, Hecht FM, Stewart A (2009). Body awareness: construct and self-report measures. PLoS One.

[28] Price CJ, Thompson EA (2007). Measuring dimensions of body connection: body awareness and bodily dissociation. J Altern Complement Med.

[29] Pöhlmann K, Roth M, Brahler E, Joraschky P (2014). The Dresden body image inventory (DKB-35): validity in a clinical sample. Psychother Psychosom Med Psychol.

[30] Pöhlmann K, Thiel P, Joraschky P, Joraschky P, Lausberg H, Pöhlmann K (2008). Development and validation of the Dresden body image questionnaire. Body oriented diagnostics and psychotherapy in patients with eating disorders.

[31] Scheffers M, van Duijn MAJ, Bosscher RJ, Wiersma D, Schoevers RA, van Busschbach JT (2017). Psychometric properties of the Dresden body image questionnaire: a multiple-group confirmatory factor analysis across sex and age in a Dutch non-clinical sample. PLoS One.

[32] Abbott BD, Barber BL (2010). Embodied image: gender differences in functional and aesthetic body image among Australian adolescents. Body Image.

[33] Davison Tanya E., McCabe Marita P. (2005). Relationships Between Men’s and Women’s Body Image and Their Psychological, Social, and Sexual Functioning. Sex Roles.

[34] Tylka TL, Wood-Barcalow NL (2015). The body appreciation Scale-2: item refinement and psychometric evaluation. Body Image.

[35] Algars A, Santtila P, Varjonen M, Witting M, Johansson A, Jern P (2009). The adult body: how age, gender, and body mass index are related to body image. J Aging Health.

[36] Sack M, Boroske-Leiner K, Lahmann C (2010). Association of nonsexual and sexual traumatizations with body image and psychosomatic symptoms in psychosomatic outpatients. Gen Hosp Psychiatry.

[37] Van der Boom KJ, Houtveen JH (2014). Psychiatric comorbidity in patients in tertiary care suffering from severe somatoform disorders. Tijdschrift voor psychiatrie.

[38] Brown TA (2015). Confirmatory factor analysis for applied research.

[39] Gregorich SE (2006). Do self-report instruments allow meaningful comparisons across diverse population groups? Testing measurement invariance using the confirmatory factor analysis framework. Med Care.

[40] Rusticus SA, Hubley AM, Zumbo BD (2008). Measurement invariance of the appearance schemas inventory-revised and the body image quality of life inventory across age and gender. Assessment.

[41] Satorra A, Bentler PM, Eye AV, Clogg CC (1994). Corrections to test statistics and standard errors in covariance structure analysis. Latent variables analysis: applications for developmental research.

[42] Muthén LK, Muthén BO (2007). Mplus User’s Guide.

[43] Field AP (2009). Discovering statistics using SPSS.

[44] Cohen J (1992). A power primer. Psychol Bull.

[45] Ho DE, Imai K, King G, MatchIt SEA (2011). Nonparametric preprocessing for parametric causal inference. J Stat Softw.

[46] Jacobson NS, Truax P (1991). Clinical significance: a statistical approach to defining meaningful change in psychotherapy research. J Consult Clin Psychol.

[47] Schulte-van Maaren YW, Giltay EJ, van Hemert AM, Zitman FG, de Waal MW, Van Rood YR (2014). Reference values for the body image concern inventory (BICI), the whitely index (WI), and the checklist individual strength (CIS-20R): the Leiden routine outcome monitoring study. J Affect Disord.

[48] Scheffers M, van Busschbach JT, Bosscher RJ, Aerts LC, Wiersma D, Schoevers RA (2017). Body image in patients with mental disorders: characteristics, associations with diagnosis and treatment outcome. Compr Psychiatry.

[49] Scheffers M, Hoek M, Bosscher RJ, van Duijn MAJ, Schoevers RA, van Busschbach JT (2017). Negative body experience in women with early childhood trauma: associations with trauma severity and dissociation. Eur J Psychotraumatol.

[50] Scheffers M, van Duijn MAJ, Beldman M, Bosscher RJ, van Busschbach JT, Schoevers RA (2019). Body attitude, body satisfaction and body awareness in a clinical group of depressed patients: an observational study on the associations with depression severity and the influence of treatment. J Affect Disord.

[51] Houtveen JH, van Broeckhuysen-Kloth S, Lintmeijer LL, Bühring MEF, Geenen R (2015). Intensive multidisciplinary treatment of severe somatoform disorder. A prospective evaluation. J Nerv Ment Dis.

[52] Rohricht F (2015). Body psychotherapy for the treatment of severe mental disorders - an overview. Body Mov Dance Psychother.

[53] Price C, Mehling WE, Thompson DL, Brooks M (2016). Body awareness and pain. Integrative pain management.

[54] Schaefer M, Egloff B, Witthoft M (2012). Is interoceptive awareness really altered in somatoform disorders? Testing competing theories with two paradigms of heartbeat perception. J Abnorm Psychol.

[55] van der Maas LC, Koke A, Pont M, Bosscher RJ, Twisk JW, Janssen TW (2015). Improving the multidisciplinary treatment of chronic pain by stimulating body awareness: a cluster-randomized trial. Clin J Pain.

[56] Körperschema LH, Joraschky P, Loew T, Röhricht F (2009). Körperbild und Bewegungsmuster - Bewegungsanalyse in der Diagnostik von Körperschema und Körperbildstörungen. Körpererleben und Körperbild. Ein Handbuch zur Diagnostik.

[57] Derogatis LR (1994). SCL-90-R: symptom Checklist-90-R.

[58] Porcelli P, Guidi J (2015). The clinical utility of the diagnostic criteria for psychosomatic research: a review of studies. Psychother Psychosom.

[59] Prins MA, Woertman L, Kool MB, Geenen R (2006). Sexual functioning of women with fibromyalgia. Clin Exp Rheumatol.

[60] de Boer MK, Castelein S, Bous J, van den Heuvel ER, Wiersma D, Schoevers RA (2013). The antipsychotics and sexual functioning questionnaire (ASFQ): preliminary evidence for reliability and validity. Schizophr Res.

