# Supplementary material for: Body image in patients with somatoform disorder
Source: BMC Psychiatry. 2018 Oct 22;18:346. doi: 10.1186/s12888-018-1928-z (PMC6198536; doi:10.1186/s12888-018-1928-z)
Supplement: Supplementary file 1 — Table S1. Item means and standard deviations of the DBIQ items* in SFD sample grouped per subscale. (DOCX 22 kb) [file 12888_2018_1928_MOESM1_ESM.docx]

| **Table S1.** Item means and standard deviations of the DBIQ items* in SFD sample grouped per subscale | | |
| --- | --- | --- |
| **Vitality** | **Mean** | ***SD*** |
| 2. I often feel physically run down (R) | 2.15 | 1.04 |
| 3. I lack energy and motivation (R) | 2.92 | 1.18 |
| 6. I often feel physically exhausted (R) | 2.39 | 1.14 |
| 8. I am physically fit | 1.82 | 1.05 |
| 14. I have lots of energy | 1.84 | 1.05 |
| 17. I am in good physical condition | 2.12 | 1.05 |
| 26. I quickly reach my physical limits (R) | 2.15 | 1.16 |
| 32. I am physically strong and resilient | 2.34 | 1.17 |
| **Body acceptance** |  |  |
| 7. There are lots of situations in which I feel happy about my body | 2.55 | 1.19 |
| 12. I like my body | 2.60 | 1.20 |
| 15. I choose clothing that hides the shape of my body (R) | 3.61 | 1.28 |
| 18. I often feel uncomfortable about my body (R) | 2.90 | 1.25 |
| 23. I wish I had a different body (R) | 3.18 | 1.53 |
| 25. I am satisfied with my appearance | 3.27 | 1.21 |
| 28. If I could change something about my body, I would do it (R) | 2.96 | 1.53 |
| **Self-aggrandizement** |  |  |
| 1. I move gracefully | 2.06 | 1.00 |
| 10. Other people find me attractive | 2.86 | 0.99 |
| 13. I find it pleasant and exhilarating when someone looks at me attentively | 2.56 | 1.20 |
| 20. I feel more valued when someone pays attention to my body | 2.79 | 1.11 |
| 29. My body is expressive | 2.62 | 1.09 |
| 31. I use my body to attract attention | 1.45 | 0.75 |
| 33. I like showing my body | 1.91 | 0.96 |
| 34. I like to be the centre of attention | 1.85 | 0.96 |
| **Sexual fulfilment** |  |  |
| 4. I experience intense and pleasurable feelings during sex | 2.53 | 1.33 |
| 9. I am very satisfied with my sexual experiences | 2.45 | 1.38 |
| 16. I think sex is an important part of life | 2.50 | 1.21 |
| 21. I am able to lay aside my inhibitions in sexual situations | 2.53 | 1.36 |
| 27. I am able to enjoy my sexuality | 2.62 | 1.09 |
| 35. My sexual experiences are satisfying | 2.65 | 1.40 |
| **Physical contact** |  |  |
| 5. Physical contact is important for me to express closeness | 3.25 | 1.23 |
| 11. I look for physical intimacy and affection | 2.67 | 1.20 |
| 19. I do not like people touching me (R) | 3.59 | 1.14 |
| 22. I like it when people put their arms around me | 3.44 | 1.12 |
| 24. I consciously avoid touching other people (R) | 3.84 | 1.13 |
| 30. I only allow a few people to touch me (R) | 2.88 | 1.29 |

* items were translated from German into English using forward and backward translation.

R: Reversely coded item. Recoding was performed before the mean was computed.
