# Supplementary material for: Body image in patients with somatoform disorder
Source: BMC Psychiatry. 2018 Oct 22;18:346. doi: 10.1186/s12888-018-1928-z (PMC6198536; doi:10.1186/s12888-018-1928-z)
Supplement: Supplementary file 2 — Figure S1. Age distribution of males and females across the three diagnostic categories and in the general population. (DOCX 99 kb) [file 12888_2018_1928_MOESM2_ESM.docx]

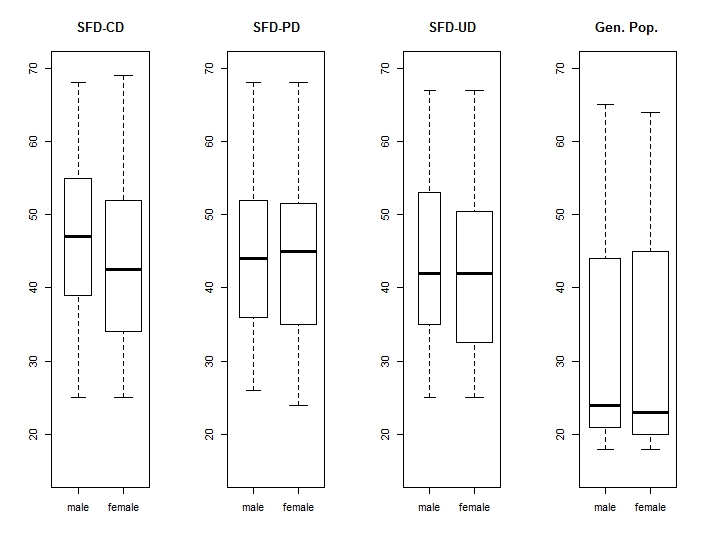


Figure S1. Age distribution of males and females across the three diagnostic categories and in the general population.

CD = Conversion Disorder; PD = Pain Disorder; UD = Undifferentiated Somatoform Disorder
