# Supplementary material for: Body image in patients with somatoform disorder
Source: BMC Psychiatry. 2018 Oct 22;18:346. doi: 10.1186/s12888-018-1928-z (PMC6198536; doi:10.1186/s12888-018-1928-z)
Supplement: Supplementary file 3 — Table S2. Measurement invariance across the control group and the somatoform group and within the somatoform group across sex. (DOCX 21 kb) [file 12888_2018_1928_MOESM3_ESM.docx]

| **Table S2** Measurement invariance across the control group and the somatoform group and within the somatoform group across sex | | | | | | | | |
| --- | --- | --- | --- | --- | --- | --- | --- | --- |
| Model | | *χ^2^* | *df* | T_s_ | RMSEA (90 % CI) | SRMR | CFI | TLI |
|  | Measurement Invariance  subjects control group and somatoform group |  |  |  |  |  |  |  |
| 1A | configural invariance | 3868 | 1100 |  | .060 (.058 -.062) | .065 | .844 | .832 |
| 1B | weak invariance | 3981 | 1130 | 74.08^**^ | .060 (.057 -.063) | .069 | .840 | .831 |
| 1C | strong invariance | 4391 | 1160 | 247.39^**^ | .063 (.061 -.065) | .077 | .818 | .814 |
| 1C-1 | partial strong item 1^a^ | 4310 | 1158 | 199.88^**^ | .062 (.060 -.064) | .076 | .823 | .818 |
| 1C-2 | partial strong item 1,7^b^ | 4223 | 1156 | 144.30^**^ | .061 (.059 - 063) | .074 | .828 | .823 |
|  | | | | | | | | |
|  | Measurement Invariance sex  within somatoform group |  |  |  |  |  |  |  |
| 2A | configural invariance | 2596 | 1100 |  | .060 (.057 -.063) | .068 | .848 | .835 |
| 2B | weak invariance | 2675 | 1130 | 70.80^**^ | .060 (.057 -.063) | .074 | .843 | .835 |
| 2C | strong invariance | 2887 | 1160 | 87.17^**^ | .062 (.060 -.065) | .079 | .824 | .820 |
| 2C-1 | partial strong item 15^c^ | 2808 | 1158 | 51.95 | .061 (.058 -.064) | .073 | .832 | .828 |
| *χ^2^* = chi square; *df* = degrees of freedom; T_s_  = Scaled Difference in Chi-Squares (SDCS) test statistic; RMSEA = Root Mean Square Error of Approximation; 90 % CI = 90 %  confidence interval of the RMSEA; SRMR = Standardized Root Mean Square Residual; CFI = comparative fit index; TLI = Tucker Lewis index.  ^a^ modification index item 1 = 84.979; ^b^ modification index item 7 = 83.041; ^c^ modification index item 15 = 55.122  **p* < .01. ** *p* < .001. | | | | | | | | |
