# Supplementary material for: Body image in patients with somatoform disorder
Source: BMC Psychiatry. 2018 Oct 22;18:346. doi: 10.1186/s12888-018-1928-z (PMC6198536; doi:10.1186/s12888-018-1928-z)
Supplement: Supplementary file 4 — Table S3. Mean (M) and standard deviations (SD) of scores on the Dresden Body Image Questionnaire (DBIQ) in patients with somatoform disorder (n = 657) and control sample (n = 761), test of the difference based on scale items deleted and effect size (Cohen’s d). (DOCX 20 kb) [file 12888_2018_1928_MOESM4_ESM.docx]

| **Table S3** Mean (M) and standard deviations (*SD*) of scores on the Dresden Body Image Questionnaire (DBIQ) in patients with  somatoform disorder (*n* = 657) and control sample (*n* = 761), test of the difference based on scale items deleted and effect size (Cohen’s *d*) | | | | | | | | |
| --- | --- | --- | --- | --- | --- | --- | --- | --- |
|  | Somatoform | Somatoform | Control sample | Control sample | Based on scale items deleted | | |  |
| (sub)scale | M (*SD*) | M (*SD*) | M (*SD*) | M (*SD*) | *t* | *df* | Cohen’s *d* |  |
| total mean score | 2.65 (0.58)  Items 1 and 7 deleted | 2.63 (0.58) | 3.67 (0.45)  Items 1 and 7 deleted | 3.66 (0.45) | 36.35* | 1233 | 1.96 |  |
| vitality | 2.22 (0.72) | 2.22 (0.72) | 3.87 (0.58) | 3.87 (0.58) | 47.31* | 1267 | 2.52 |  |
| body acceptance | 3.09 (0.96)  Item 7 deleted | 3.01 (0.95) | 3.83 (0.67)  Item 7 deleted | 3.83 (0.67) | 16.48* | 1172 | 0.89 |  |
| sexual fulfilment | 2.50 (1.02) | 2.50 (1.02) | 3.78 (0.71) | 3.78 (0.71) | 26.93* | 1145 | 1.46 |  |
| physical contact | 3.28 (0.81) | 3.28 (0.81) | 3.80 (0.60) | 3.80 (0.60) | 13.66* | 1195 | 0.73 |  |
| self-aggrandizement | 2.29 (0.66)  Item 1 deleted | 2.26 (0.64) | 3.10 (0.58)  Item 1 deleted | 3.12 (0.55) | 24.32* | 1310 | 1.30 |  |
| * *p* < .001 | | | | | | | | |
